# 1099. CMV PCR Agreement between Blood and Dried Blood Spots in Infants with Congenital Cytomegalovirus Infection

**DOI:** 10.1093/ofid/ofad500.072

**Published:** 2023-11-27

**Authors:** Juhyeong Kim, Huanyu Wang, Alexandra Katherine Medoro, Traci Pifer, Rachelle Crisan, Amy Leber, Kaitlyn Flint, Sebastian Powell, Pablo J Sanchez, Masako Shimamura

**Affiliations:** Nationwide Children's Hospital, Dublin, OH; Nationwide Children's Hospital, Dublin, OH; Nationwide Children's Hospital, Dublin, OH; Nationwide Children's Hospital, Dublin, OH; Nationwide Children's Hospital, Dublin, OH; Nationwide Childrens Hospital, Columbus, Ohio; Nationwide Children's Hospital, Dublin, OH; Nationwide Children's Hospital, Dublin, OH; Nationwide Children's Hospital - The Ohio State University, Columbus, OH; Nationwide Children's Hospital, Dublin, OH

## Abstract

**Background:**

Dried blood spot (DBS) PCR sensitivity for congenital cytomegalovirus (cCMV) screening ranges from 34% to 86%, but it is unclear whether a negative DBS PCR reflects an absence of CMV DNAemia or a technical limitation in viral detection by DBS PCR. The objective of this study was to determine the agreement between CMV DNA PCR tests in plasma and DBS among cCMV infected infants.

**Methods:**

This single center retrospective cohort study evaluated infants diagnosed with cCMV infection by urine CMV PCR at age ≤ 21 days due to clinical symptoms, failed newborn hearing screen, or NICU admission screening. Subjects with a plasma CMV PCR test performed within the first month after birth and a newborn CMV DBS PCR test were included in the study. Agreement between the plasma and DBS PCR tests was calculated using Cohen’s kappa coefficient. Clinical characteristics and viral loads were compared between groups with concordant and discordant DBS and plasma PCR tests using the Kruskal-Wallis or Mann-Whitney U test.

**Results:**

70 cCMV infected infants were included in the study. 30 (43%) were female, 51 (73%) were white, 13 (19%) were black, 6 (8%) were other/multiracial, 3 (4%) were Hispanic, and 54 (77%) had symptomatic cCMV infection. 49 (70%) had DBS+/plasma+ PCR; 1 (1.4%) had DBS+/plasma- PCR; 14 (20%) had DBS-/plasma+ PCR; and 6 (9%) had DBS-/plasma- PCR. Groups had similar distributions of sex, race, ethnicity, gestational age, birthweight, birth length, birth head circumference, and symptomatic infection. DBS sensitivity was 71%. Agreement between the tests was fair (κ = 0.348, 95% confidence interval = 0.115-0.581). Of the 20 subjects with DBS- tests, 70% had plasma+ PCR and 30% had undetectable CMV DNA in plasma. Infants with DBS+/plasma+ PCR had significantly higher plasma viral loads compared to those with DBS-/plasma+ PCR (2245 IU/ml [761-5773] vs. 321 IU/ml [165-464], median [interquartile range]) (p < 0.0001).

**Figure:**
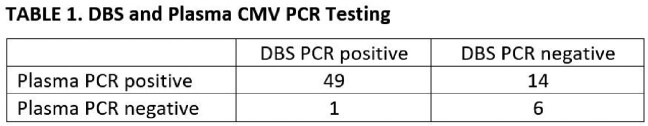

**Figure:**
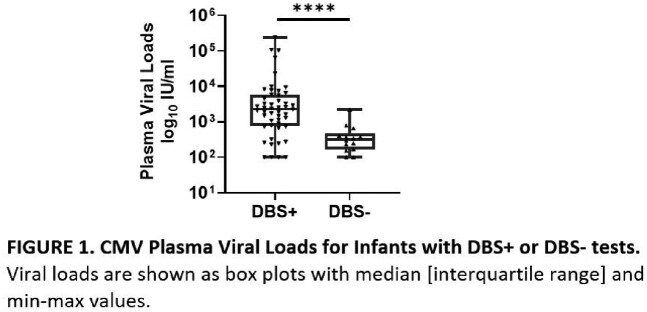

**Conclusion:**

In this predominantly symptomatic cohort, DBS PCR identified the majority (71%) of infants with cCMV infection. A negative DBS PCR was associated with either low-level plasma CMV DNAemia that was seen in 20% of cCMV infected infants, or no plasma DNAemia in 9% of infants. These findings may impact the use of DBS as a screening test for cCMV infection.

**Disclosures:**

**Alexandra Katherine Medoro, MD**, Merck: Grant/Research Support **Amy Leber, PhD**, Biomeriux: Grant/Research Support|BioRad: Advisor/Consultant|Cephied: Grant/Research Support|Diasorin: Grant/Research Support

